# Bacterial nanocellulose from agro-industrial wastes: low-cost and enhanced production by *Komagataeibacter saccharivorans* MD1

**DOI:** 10.1038/s41598-020-60315-9

**Published:** 2020-02-26

**Authors:** Deyaa Abol-Fotouh, Mohamed A. Hassan, Hassan Shokry, Anna Roig, Mohamed S. Azab, Abd El-Hady B. Kashyout

**Affiliations:** 1 Electronic Materials Researches Department, Advanced Technology and New Materials Research Institute, City of Scientific Research and Technological Applications (SRTA-City), New Borg El-Arab City, P.O. Box: 21934, Alexandria, Egypt; 2 Protein Research Department, Genetic Engineering and Biotechnology Research Institute (GEBRI), City of Scientific Research and Technological Applications (SRTA-City), New Borg El-Arab City, P.O. Box: 21934, Alexandria, Egypt; 3 Environmental Engineering Department, Egypt-Japan University of Science and Technology, New Borg El-Arab City, Alexandria Egypt; 4 Institute of Materials Science of Barcelona (ICMAB-CSIC), Campus of the UAB, 08193 Bellaterra, Spain; 5 Department of Botany & Microbiology, Faculty of Science, Al-Azhar University, Cairo, Egypt

**Keywords:** Biomaterials, Applied microbiology

## Abstract

Bacterial nanocellulose (BNC) has been drawing enormous attention because of its versatile properties. Herein, we shed light on the BNC production by a novel bacterial isolate (MD1) utilizing various agro-industrial wastes. Using 16S rRNA nucleotide sequences, the isolate was identified as *Komagataeibacter saccharivorans* MD1. For the first time, BNC synthesis by *K. saccharivorans* MD1 was investigated utilizing wastes of palm date, fig, and sugarcane molasses along with glucose on the Hestrin-Schramm (HS) medium as a control. After incubation for 168 h, the highest BNC yield was perceived on the molasses medium recording 3.9 g/L with an initial concentration of (v/v) 10%. The physicochemical characteristics of the BNC sheets were inspected adopting field-emission scanning electron microscope (FESEM), atomic force microscopy (AFM), X-ray diffraction (XRD), and Fourier transform infrared (FTIR) analysis. The FESEM characterization revealed no impact of the wastes on either fiber diameter or the branching scheme, whereas the AFM depicted a BNC film with minimal roughness was generated using date wastes. Furthermore, a high crystallinity index was estimated by XRD up to 94% for the date wastes-derived BNC, while the FTIR analyses exhibited very similar profiles for all BNC films. Additionally, mechanical characteristics and water holding capacity of the produced BNCs were studied. Our findings substantiated that expensive substrates could be exchanged by agro-industrial wastes for BNC production conserving its remarkable physical and microstructural properties.

## Introduction

In the last decades, bacterial nanocellulose (BNC) has earned increasing global interest because of its remarkable physical and chemical properties, including green processing, low production costs, elevated mechanical properties, hydrophilicity, excellent biocompatibility, and biodegradability^1,2^.

Certain gram-negative non-pathogenic bacterial genera like *Rhizobium, Xanthococcus, Pseudomonas, Azotobacter, Aerobacter, and Alcaligenes* were reported to produce nanocellulose extracellularly, but the most common BNC-producing strains belong to the genus *Komagataeibacter* (formerly *Acetobacter* or commonly acetic acid bacteria)^1^.

Bacteria produce the BNC through a process of dual coupled steps: polymerization and crystallization. In the bacterial cytoplasm, glucose residues polymerize to β-1,4 glucan linear chains where they are extracellularly secreted. The developed chains are crystallized to microfibrils, then certain numbers of microfibrils consolidate to materialize highly pure 3D porous network of entangled nanoribbons of 20–60 nm in width^3^.

Compared to plant cellulose, BNC is produced in a pure form; free of lignin, pectin, and hemicelluloses. BNC ultra-fine structure possesses much higher merits of crystallinity, higher liquid absorption capacity, higher degree of polymerization, higher specific surface area, and higher mechanical properties making it a superior choice to the plant cellulose in many applications^3,4^. Moreover, the readiness of BNC for modification renders it highly superior compared to cellulose of plant origin. BNC could be shaped during the fermentation period to devise tubes, spheres, or membranes according to the application demands^2^.

Another aspect is the abundance of hydroxyl groups in the BNC, which facilitates its functionalization or compositing with other reinforcing compounds that confer BNC with new physical properties^1^, such as antimicrobial activity^5^, electro-conductivity^6^, or even multifunctional BNC composites^7^. Thus, the application sectors of BNC are continuously broadening, including bioprocessing, biomedical and pharmaceutical applications, wastewater treatment^8^, electro-conductive materials, packaging^9^, and food industry^10^.

On the other hand, the overall production process of BNC still requires major improvement to be more competitive, primarily due to the low BNC productivity of the known strains and the use of fine and expensive culture medium constituents. It is worth pointing out that the culture medium comprises approximately 30% of the total BNC production cost^11^. Therefore, one of the main reported confrontations was to totally or partially replace the pricy medium components with new low-cost ones that could promote the BNC yield within short time periods^12,13^. Towards this aim, intensive studies have been conducted utilizing waste stream and agricultural wastes for obtaining high yields of BNC, demonstrating how the properties of the produced BNC might be altered according to the medium constituents, culture conditions and/or the BNC producer^12,14^.

Hence, we report on the exploration of a novel and high potent BNC-producing bacterial isolate, *Komagataeibacter saccharivorans*, which are able to utilize the hydrolysates of three cheap agro-industrial wastes (palm date fruits, fig fruits, and sugarcane molasses) for cost-effective BNC production. The impact of these treated wastes on the physical and structural properties of the produced BNC is investigated employing a series of characterization instruments. Gopu and Govindan^15^ isolated the strain *Komagataeibacter saccharivorans* BC1 and ascertained mannitol as the best carbon source for BNC production. To the best of our knowledge, the present study is the first report probing the BNC productivity using *Komagataeibacter saccharivorans* utilizing these wastes.

## Results and Discussion

The global demands to upcycling wastes for producing value-added products become an imperative not only for the economic viewpoint, but also from the waste reduction perspective and the implementation of higher green standards to the agriculture and food processing industries.

Many agricultural and industrial wastes have been already investigated for the BNC production, including cashew tree residues^16^, dry olive mill residues^17^, konjac powder^18^, rice bark^19^, waste beer yeast^20^, oat hulls^21^, and coffee cherry husk^22^. However, there is still substantial interest to find out and utilize economical substrates that could promote the yield of BNC.

Egypt is the largest producer of palm date fruits over the world, and second largest fig producing country as well^23^. The processes of harvesting, packing, transporting, storing, and marketing of these fruits generate considerable amounts of unsold low-quality fruits, which are usually discarded as wastes. Being considered debris does not rule out their richness of nutritional contents, which can be exploited as substrates for synthesizing several unique and worthy microbial products; for instance, BNC^14^. On the other hand, molasses is a common by-product of the sugar industry, which was extensively studied as cheap carbon sources for BNC production as well^24–26^.

### Isolation and identification of BNC-producing strain

**Figure 1:**
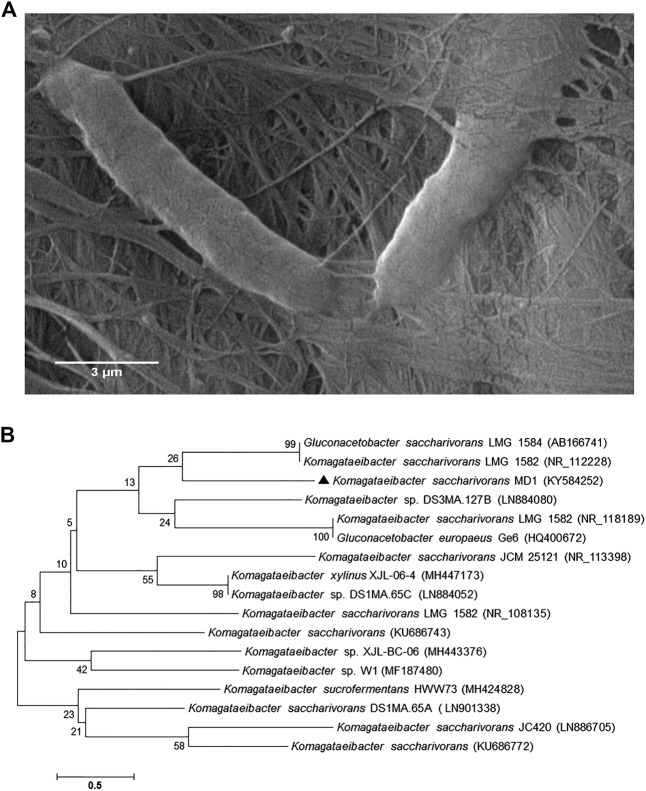
(**A**) FESEM of BNC shelters the rod bacterial isolate MD1, and (**B**) Phylogenetic tree of *Komagataeibacter saccharivorans* MD1 showing its position within the closest strains based on the nucleotide sequences of 16S rRNA gene. The accession numbers of each 16S rRNA nucleotide sequences deposited in GenBank database are shown in parentheses.

In the current study, our isolation plan unraveled two BNC positive cultures; one was isolated from fermented beverages and designated as (MD1), while the other was purified from table vinegar remnant and denominated as (VB3). Further evaluations nominated the dominant isolate (MD1) to undergo advanced inspections as the highest BNC-producing isolate reached to 2.6 g/L. The isolate (MD1) underwent a set of morphological and physiological examinations to determine its phenotypical features as presented in Table S1. Furthermore, the FESEM analysis at a magnification of 30000 x illustrated the rod cells of isolate MD1 harboring the network structure of the synthesized BNC as shown in Fig. 1A. Moreover, the genotypic characterization of the isolate MD1 was performed by amplifying the 16S rRNA and the size of the obtained fragment was about 1500 bp. The 16S rRNA nucleotide sequences of 1294 bp were obtained using the 16S rRNA forward and reverse primers. The homologous sequences were retrieved via the Nucleotide Basic Local Alignment Search Tool (BLASTn)^27^ and the results exhibited 100% similarity to various *Komagataeibacter saccharivorans* strains such as *Komagataeibacter saccharivorans* LMG 1582 (NR_118189), and *Komagataeibacter saccharivorans* JCM 25121 (NR_113398). Accordingly, the isolate MD1 was identified as *Komagataeibacter saccharivorans* MD1, and deposited in The National Center for Biotechnology Information **(**NCBI) GenBank under the accession number KY584252 (https://www.ncbi.nlm.nih.gov/nuccore/KY584252). Figure 1B demonstrates the phylogenetic tree of the 16S rRNA including the position of *Komagataeibacter saccharivorans* MD1 compared to the available sequences on NCBI GenBank database.

### Production of BNC on wastes

**Figure 2:**
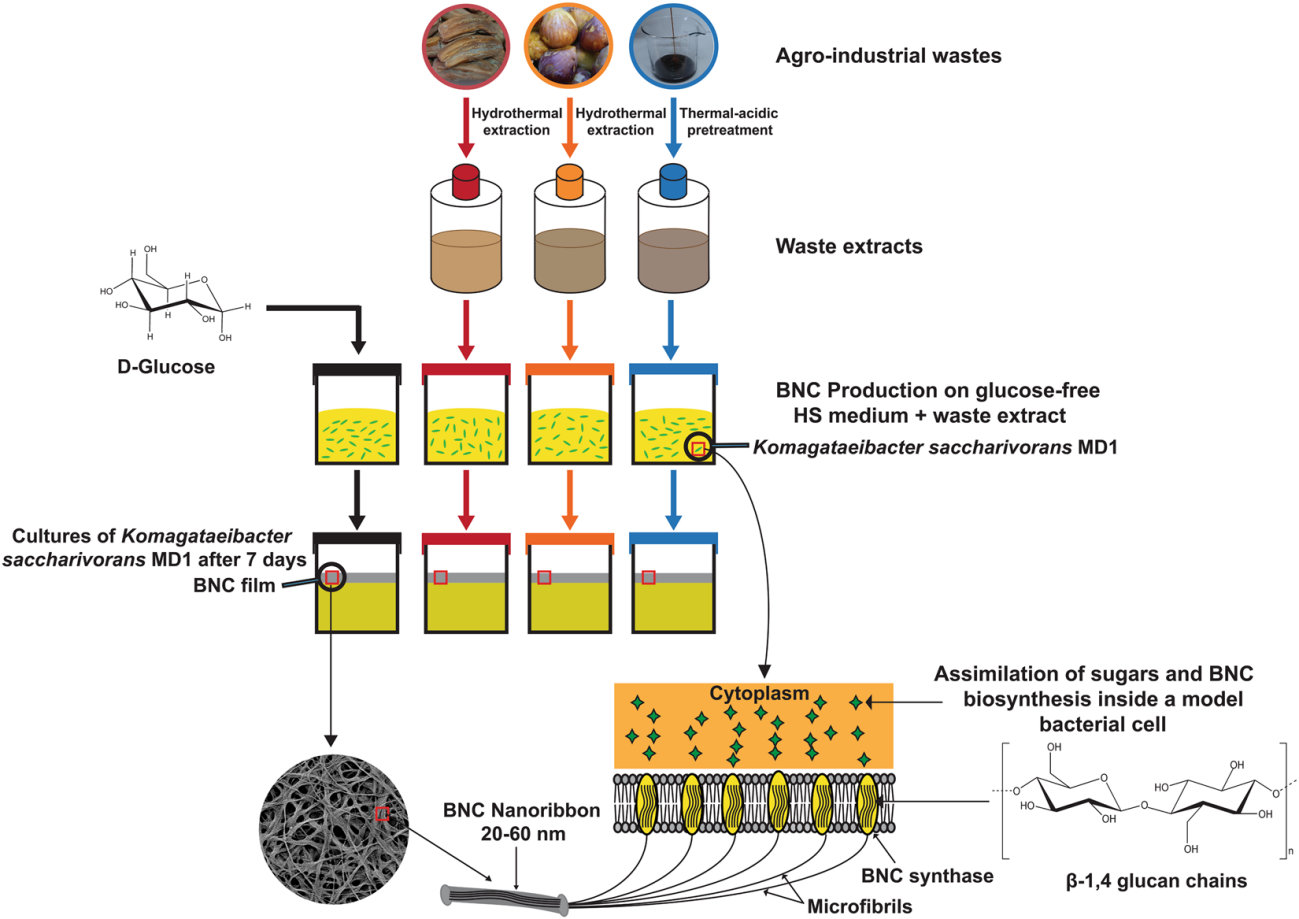
Schematic diagram demonstrates the series of steps from the pre-treatment of wastes to supplement the (HS) medium for BNC biosynthesis by *K. saccharivorans* MD1 and the internal fabrication of BNC within the bacterial cells.

**Figure 3:**
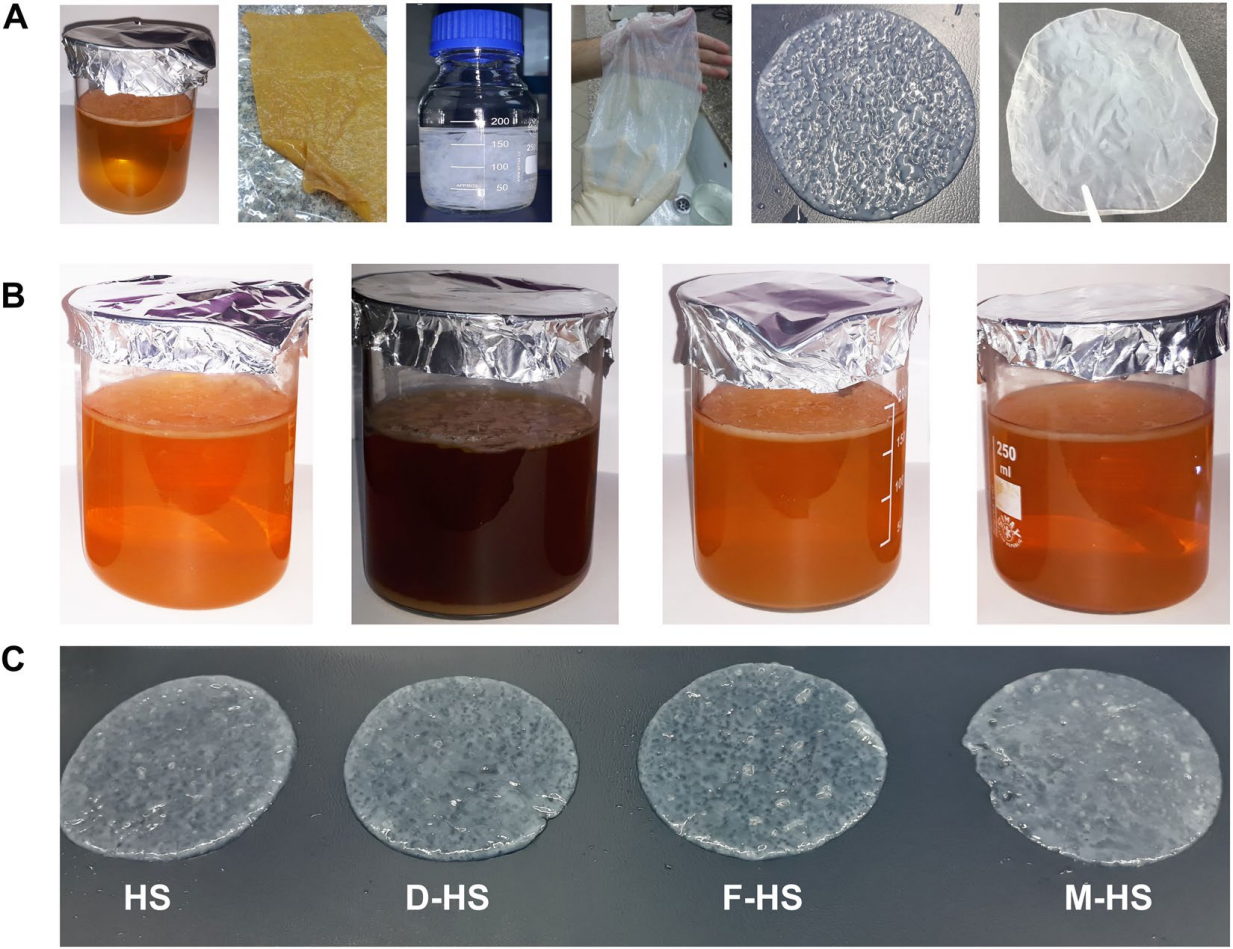
(**A**) The culture of *K. saccharivorans* MD1 after 10 days and the general morphology of the produced BNC film illustrating the treatment procedures of films until the final product, (**B**) BNC production on (HS), (D-HS), (F-HS), and (M-HS) media, and (**C**) is the corresponding generated BNC films, respectively.

We could statically produce BNC by *K. saccharivorans* MD1 on (HS) media as a control along with the three media formulated by replacing the glucose in the (HS) with the extract of date fruit wastes (E-DFW), extract of fig fruit wastes (E-FFW), and the treated sugarcane molasses (T-SCM) as indicated in Fig. 2. This blueprint pinpoints the implemented methods in the current research to produce the BNC indicating the assimilation of sugars within the bacterial cells and the formulation of β-1,4 glucan chains to finally construct the microstructure of cellulose. Indeed, the BNCs generated on the three wastes primarily showed comparable morphological features to the control BNC, particularly after washing **(**Fig. 3**)**.

**Figure 4:**
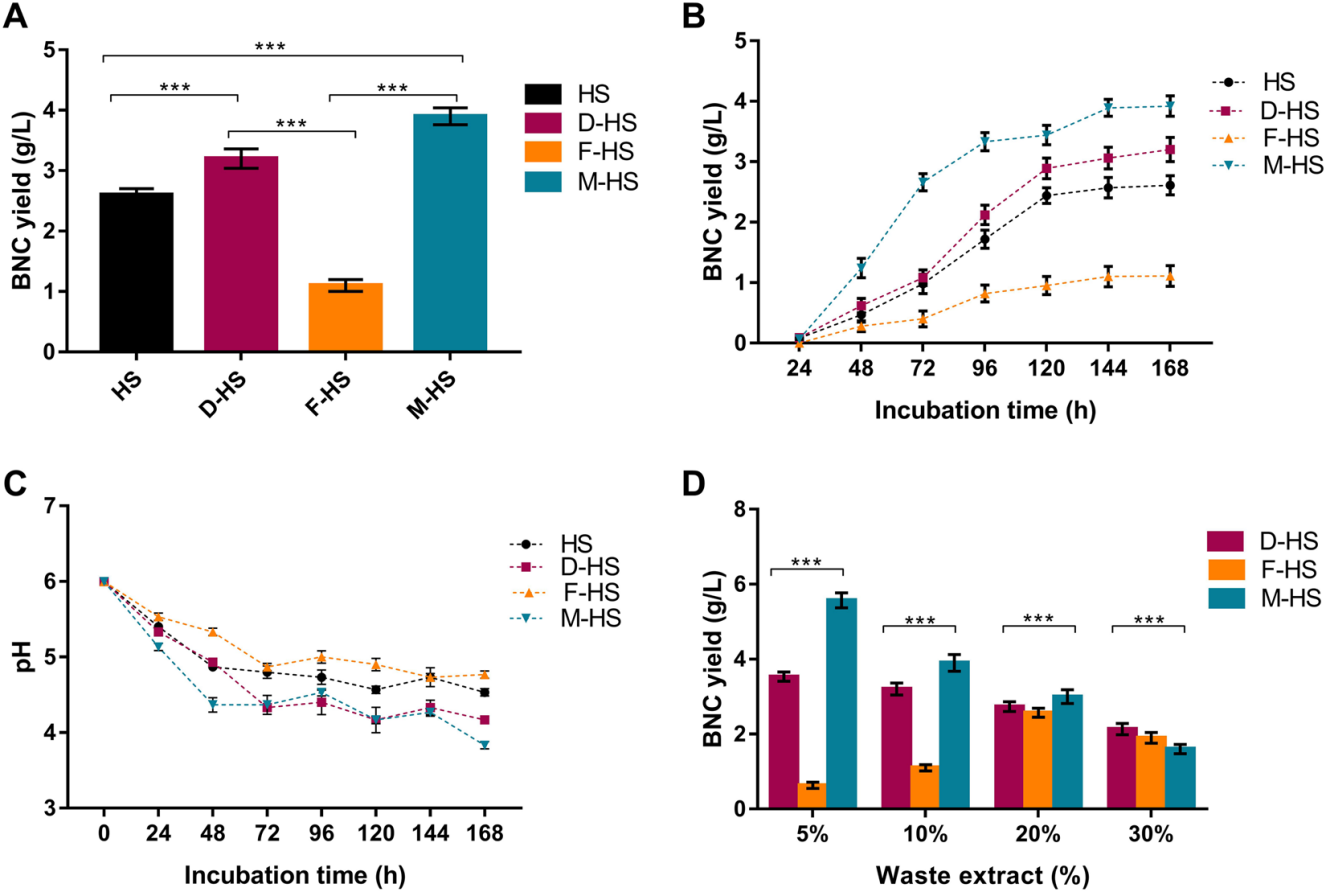
(**A**) The BNC dry yield from *K. saccharivorans* MD1 utilizing the four substrates after incubation period of 168 h, (**B**) effect of incubation time on BNC production by *K. saccharivorans* MD1, (**C**) monitor of pH values during the BNC production by *K. saccharivorans* MD1, and (**D**) impact of different initial concentrations of the date, fig, and molasses substrates on the yield of BNC. Values express means ± SD and ^***^P < *0.001* for the multiple comparison when n = 6.

Figure 4A depicts that after 168 h of bacterial cultures incubation, the treated molasses medium (M-HS) exhibited the highest BNC productivity yielding 3.9 g/L, followed by the medium complemented with the date extract (D-HS) recording 3.2 g/L. Both media enhanced the BNC production compared to the (HS) medium that yielded 2.6 g/L dry weight of BNC. Moreover, out of the four media, the (M-HS) exhibited the highest conversion ratio (α = 91%), which was higher with two points than the ratio shown by the (HS) media (α = 89%) that contains the more pricy fine D-glucose as a carbon source **(**Table 1**)**.

**Table 1:** BNC production rates and yields utilizing the three media supplemented with the date, fig, and molasses substrates comparing to yield on (HS) medium by *K. saccharivorans* MD1 after incubation for 168 h.

Medium	Si (g/L)	M (g/L)	∆S (g/L)	α (%)	Y (%)	R (g/L.h)
HS	20	2.6	17.8	89	14.6	0.01
D-HS	56	3.2	44.6	79	5.7	0.019
F-HS	14	1.1	10.3	73	7.8	0.006
M-HS	62	3.9	57	91	6.2	0.02

(Si) Initial substrate concentration; (M) amount of BNC produced; (∆S) amount of the consumed substrate; (α) substrate conversion ratio; (Y) BNC production yield; (R) BNC production rate or productivity.

The thermal-acidic pre-treatment was proposed to improve the properties of the molasses, raise its (glucose-fructose) contents per volume, and eliminate most of chemicals that might hinder the microbial growth or affect the product yield^28,29^. Bae and Shoda^24^ elucidated the role of thermal acidic pre-treatment of molasses in almost full degradation of the included sucrose to its initial precursors: glucose and fructose. They reported that cultivation of the strain *Acetobacter xylinum* BPR2001 on the pre-treated molasses augmented the BNC yield by 76%, comparing to the yield on crude molasses. Meanwhile, the strain *Komagataeibacter rhaeticus* yielded 4.01 g/L as a consequence of growing on a blend of glucose and crude sugarcane molasses as an ideal carbon source at concentrations of 30 and 20 g/L, in respective order^26^.

Rodrigues *et al*.^30^ reported the greatest BNC production, about 7.5 g/L, by the strain *Komagataeibacter xylinus* BPR 2001 after cultivation for 9 days on a mixture of sugarcane molasses (5.38%, m/v) and ethanol (1.38%, v/v).

Date fruit and date syrup revealed high contents of glucose and fructose, in addition to naturally existing minerals (Se, Cu, Ca, K, Mg, Mn, Zn)^31,32^. Besides being one of the main energy sources for the metabolic machinery, glucose is the building block of the cellulose polymer; therefore, D-glucose is the main constituent of most synthetic reported media for BNC production^33–35^. Additionally, a previous study pointed out that fructose represents an ideal carbon source for BNC production as glucose^36^. Accordingly, introducing both of them with high amounts might explain the higher BNC harvested from date wastes compared to the BNC production on (HS) medium that contains 20 g/L of initial glucose concentration. Furthermore, Keshk^36^ corroborated that the yield of BNC increased in the presence of mixed sugars comparing to the glucose as a sole carbon source.

The medium amended by fig extract (F-HS) presented the lowest initial sugar concentration (14 g/L), and BNC production in turn. However, in terms of sugar to BNC conversion yield Y (%), the (F-HS) medium exhibited the second highest BNC yield (Y = 7.8%), after the (HS) medium yield.

As can be observed in Fig. 4B, *K. saccharivorans* MD1 showed augmentation in BNC production until 120 h before the rate slows down at the last 48 h. Hence, we might presume that BNC generation rate reduces with the plummeting of nutrients, the accumulation of the organic acids, and other by-products in the culture. The produced floating nanocellulose pellicle itself might obstruct the medium aeriation that compensate the O_2_ consumed since the culture had been launched, and the nanocellulose mat secure oxygen demands exclusively for the cells entrapped in its matrix^37^. Accordingly, the cells beneath the nanocellulose mat turn out dormant, and they were quashed from participation in the BNC fabrication^13^. Throughout 168 h of incubation, the four cultures media demonstrated declining in pH rate profiles, and the pH values of the cultures (M-HS) and (D-HS) were dramatically decreased at the time end point pinpointing 3.8 and 4.1, respectively **(**Fig. 4C**)**.

The pH profiles of the four cultures emphasized the significance of investigating the optimum initial carbon source concentration for the maximum BNC yield. Figure 4D elucidates how the elevated initial wastes concentrations influence BNC production.

The decrease in the initial concentrations of the date fruit extract and the treated molasses to (v/v) 5% was effective to elevate the BNC productivity to 3.5 and 5.5 g/L, respectively. On the other hand, the same proportion of the fig extract was not supportive for the BNC production, and the improved BNC productivity on fig extract showed with an initial concentration of (v/v) 20% recording 2.5 g/L, before the BNC productivity declined again at (v/v) 30% proportion.

Considering the sugar-content richness of the utilized date extract and the treated molasses comparing to the fig extract **(**Table 1**)**, it is clearly recognized that BNC production depends linearly with the initial sugar concentration up to certain level, 5% for the date extract and molasses, and 20% for the fig extract, where an excess of sugar contents impairs the BNC production.

We suppose that the best BNC production is a compromise between the best sugar concentration and the resulted medium pH. For instance, acetic acid bacteria assimilate glucose monomers as building blocks to construct nanocellulose fibers, in addition to burn glucose units through various metabolic pathways to derive energy and keep proliferation. As a result of all these biochemical processes, gluconic acid is generated as the most abundant by-product. Therefore, the higher initial glucose concentration, the higher gluconic acid production, and its excess resulted in significant reduction of BNC productivity as the medium pH becomes extremely acidic^38^.

### Microstructure by (FESEM) investigation

**Figure 5:**
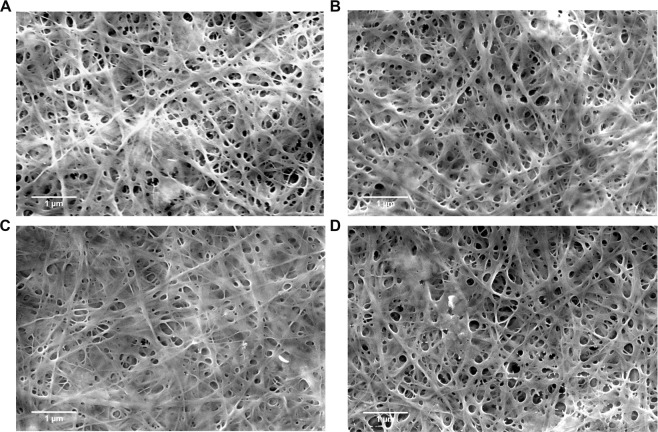
FESEM for the BNC produced by *K. saccharivorans* MD1 on (**A**) HS, (**B**) D-HS, (**C**) F-HS, and (**D**) M-HS media.

Many reports explained the influence of the culturing parameters such as the acting bacterial strain, temperature, incubation state, incubation time, or medium constituents on the properties of the produced BNC^39–43^. Recently, when studying non-conventional media in BNC production, it was found out that the chemical composition of the produced BNC was not much affected; however, the physical properties like water holding capacity, tensile, polymerization degree, and crystallinity index might be influenced by the utilized carbon source^44^. Thus, the relationship between BNC properties and medium carbon source has been drawled great attentions to get careful inspections as long as the produced BNC might be implemented in diverse applications^16,45,46^. In the current case, FESEM investigations for the four BNC fabrics were carried out. Figure 5 shows the typical tridimensional nanofibrous network distinguishing the BNC. Furthermore, FESEM analyses indicated no major variations could be observed in the dimensions of the nanofibers of the four produced BNCs, or the branching scheme of the fibrous network. The diameter of the nanofibers of the four produced BNCs scales in the range of 10–90 nm, almost in a similar manner.

We suppose that the inconsiderable impact of the utilized wastes on the microstructural and morphological features of the produced BNC nanofibers could be attributed to the efficient elaboration of the utilized extracts that made them effective substitution for D-glucose. Through growing of *K. saccharivorans* MD1 on the three media, (D-HS), (F-HS), and (M-HS), glucose and fructose are the predominant carbon sources, where they were assimilated by the cells of *K. saccharivorans* MD1 for cells propagation and to generate BNC simultaneously. The other constituents of the utilized extracts such as phytocompounds, antioxidants, and minerals may affect the physical properties of the produced BNC, but they seem to have mild or no impact on the diameter of the nanofibers or their branching scheme.

### Water holding capacity and water release rate

Water holding capacity (WHC) and water release rate (WRR) are substantial physical respects for BNC pellicles, particularly when the BNC is intended for biomedical applications such as wound dressing and tissue engineering. BNC, which is a hydrogel, retains large amounts of water mainly because of its elevated porosity and surface area per mass unit^47^.

**Figure 6:**
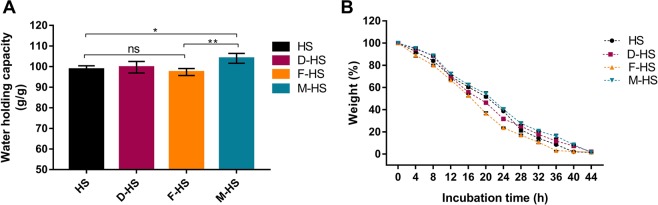
(**A**) Water holding capacity (WHC) of the BNC films produced on (HS), (D-HS), (F-HS), and (M-HS) media. (**B**) Water release rate (WRR) of the BNC films produced on (HS), (D-HS), (F-HS), and (M-HS) media. Data are presented as means ± SD. (ns) indicates non-significant, where *P* > *0.05*, while ^*^P < *0.05*, and ^**^P < *0.01* for the multiple comparison when n = 6.

The WHC of the BNC films produced on (HS), (D-HS), (F-HS), and (M-HS) media were ascertained as depicted in Fig. 6A, where the BNC fabricated on the M-HS exerts the highest WHC, recording 104 g/g. The lowest water load was found by the F-HS product, recording 97.3 g/g. Both the BNCs of the HS and D-HS exhibited comparable water capacity, with 98.6 and 99.6 g/g, respectively. This behavior implies that the WHC profiles are in accordance with the FE-SEM inspection, and corroborate our assumption of the low impact of the utilized agro-industrial wastes on the size of fibers and pores or the fibrous branching scheme.

On the other hand, the WRRs of the four BNC products throughout 48 h configure the speed, by which, the water evaporates from the films. Figure 6B demonstrates that the BNCs produced on M-HS and D-HS has the lowest WRR, where they needed 44 h to completely evaporate the water content. Meanwhile, the BNC product of HS medium could retain water until 40 h, while no further water evaporation from the BNC of the F-HS after only 36 h was observed.

The ability of BNC to load water regarded mainly to the hydrogen bonds of the forming glucan chains. Rebelo *et al*.^48^ deduced a mathematical model to elucidate the BNC water evaporation process. They proposed that the “BNC drying reaction” takes place on two steps: evaporation of the surface water as a physical process depending on the temperature, humidity, and air velocity; then heat transfer and unbinding of internal water molecules because of the breakage of hydrogen bonds per time unit.

### Surface topography by (AFM) analysis

**Figure 7:**
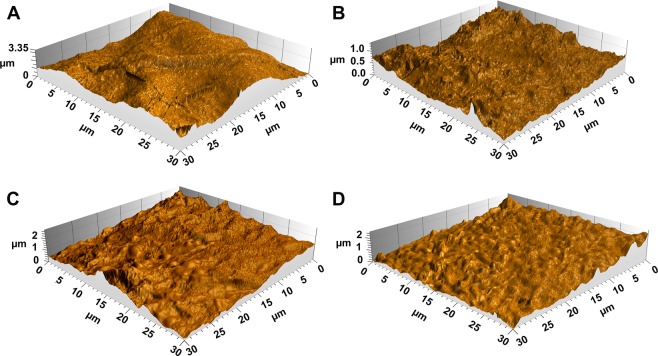
AFM 3D view depicting the surfaces of the BNC produced by *K. saccharivorans* MD1 on (**A**) HS, (**B**) D-HS, (**C**) F-HS, and (**D**) M-HS media.

Surface roughness is a crucial feature for validating BNC in many electronic devices^49,50^ and biomedical implementations^51^. The (AFM) in tapping mode was employed to study the topography of the surface area of 30 ×30 µm for the four BNC products (Fig. 7). Out of the four BNC fabrics, the highest (Rq) value was for the BNC produced on the control (HS) medium recording 0.67 µm. The BNC produced on (D-HS) formulation exhibited the smoothest surface morphology with Rq = 0.19 µm approximately less than one third of that of the control BNC product. Both BNC products of the (F-HS) and (M-HS) media showed approximate (Rq) values of 0.44 and 0.48 µm, respectively. Thus, the roughness of the four BNC products could be ordered as follows (higher roughness first): HS > M-HS > F-HS > D-HS. Fig. S1 and Table S1 correspondingly exhibit the results for the BNCs surface roughness.

### Crystalline structure by XRD analysis

BNC is a semi-crystalline biopolymer mainly composed of cellulose I, that comprises I_α_ and I_β_ polymorphs in certain proportions, which differ in correspondence to the production status^43^. In all cases, the I_α_/I_β_ ratio found to be non-correlated with the crystallinity index of any cellulose sample^52^.

**Figure 8:**
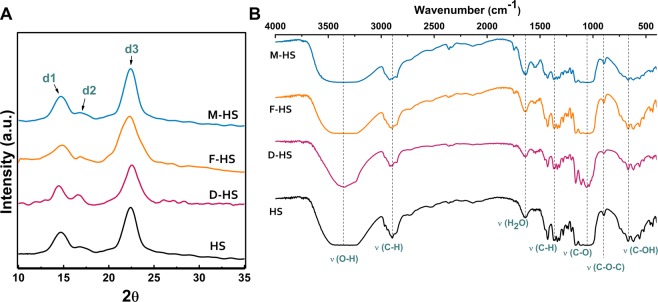
X-ray diffraction patterns (**A**) and FTIR spectra (**B**) of the BNC produced by *K. saccharivorans* MD1 on (HS), (D-HS), (F-HS), and (M-HS) media. In XRD figure, the three main peaks are indicated as d1, d2, and d3, while FTIR spectra display the corresponding assignments for all the bands.

The X-ray diffractograms of the BNCs produced on the four media indicated the emergence of three main peaks; d1, d2, and d3 as given in Fig. 8A. The peak d1 appeared at 2Ɵ = 14.66° ± 0.2° as an average of the peaks of the four BNC products, and assigned to (100) plane of I_α_ or to the plane (1–10) of I_β_. The peak d2 is detected at 2Ɵ = 16.7° ± 0.2°, and attributed to (010) plane of I_α_ or (110) plane of I_β_. The d3 at 2Ɵ = 22.4° ± 0.1°, and could be a contribution of (110) plane of I_α_, the (200) plane of cellulose I_β_^43^.

The BNC produced on the (D-HS) medium revealed the highest CrI (94%), which was higher than that of the control (HS) medium (87%). The crystallinity index of the BNC produced on (F-HS) and (M-HS) were 81% and 84%, respectively. The estimated crystallite sizes (CrS) and inter-planar distances or d-spacing (*d*) for each peaking crystallite are illustrated in Table 2.

**Table 2:** *d*-spacing (*d*), Crystallite sizes (CrS), and Crystallinity indices (CrI) of the four BNC sheets synthesized by *K. saccharivorans* MD1 culturing on (HS), (D-HS), (F-HS), and (M-HS) media.

Medium	d1		d2		d3		CrI (%)
	*d* (nm)	CrS (nm)	*d* (nm)	CrS (nm)	*d* (nm)	CrS (nm)	
HS	0.61	3.5	0.53	3.4	0.39	4.2	87
D-HS	0.61	5.4	0.53	5.5	0.39	4.1	94
F-HS	0.59	2	0.5	3.8	0.39	2.7	81
M-HS	0.6	3.6	0.52	4.3	0.39	4.3	84

Interestingly, we observed some variations in the crystallite sizes of each BNC product proposing a direct impact of the media on the BNC crystals. The crystallite size of the BNC produced on the (D-HS) medium was the greatest (5.5 nm), while that produced on (F-HS) medium showed the smallest size among the four products, recording (2 nm), postulating that some chemical substances included within the fig waste extract could interfere the assembly of chains in the corresponding BNC crystallite.

### FTIR analysis

FTIR profiles of the produced BNCs by *K. saccharivorans* MD1 on the media supplemented with the three wastes revealed identical chemical composition to the BNC control (Fig. 8B). As demonstrated in the four FTIR spectra, the wide peak around 3352 cm^−1^ is assigned to the O-H stretching, while the peak around 2900 cm^−1^ is related to C-H stretching^53^. The water bending vibrations peaked at 1639 cm^−1^, while the peaks around 1357 cm^−1^ indicate the C-H bending. Many peaks appeared between 1055-1049 cm^−1^ are corresponding to C-O stretching at C3; C-C stretching; and C-O stretching at C6. A band at 896 cm^−1^ is attributed to as C-O-C stretching at β (1,4) glycosidic linkage. Single peak of out of plan bending C-O-H appeared at 667 cm^−1^ ^54,55^. The entire band assignments are detailed in Table 3. As a consequence of these measurements, it could be inferred that the production of BNC utilizing the selected three wastes showed no impact on their chemical features, which might play an important role over the application phase. The FTIR spectra of the four BNC products demonstrated no clues for existence of cellulose II, and the whole peaks came in complete accordance with the cellulose I profile as reported by Oh *et al*.^56^ suggesting that the hydrothermal alkaline washing protocol is mild enough not to convert cellulose I to cellulose II.

**Table 3:** FTIR peak assignments of the BNC produced by *K. saccharivorans* MD1 utilizing (HS), (D-HS), (F-HS), and (M-HS) media.

Band assignment	Wavenumber (cm^−1^)			
	HS	D-HS	F-HS	M-HS
O-H stretching	3416-3244	3352	3479-3217	3460-3234
C-H stretching	2899	2906	2902	2912
water bending vibrations	1639	1639	1639	1639
C-H bending	1356	1357	1357	1359
C-O at C3; C-C stretching; and C-O at C6	1053	1055	1051	1049
C-O-H out of plan bending	667	667	667	667

### Mechanical properties

The BNC films produced on the media HS, D-HS, F-HS, and M-HS were characterized for their stress-strain performances. Table 4 summarizes the values of tensile (MPa), elongation at break (%), and Young’s modulus for the four BNC products. The BNC films generated on (D-HS) medium showed both the highest tensile and Young’s modulus with 57 ± 6 and 17 ± 4 MPa, respectively. Meanwhile, the BNC purified from the (HS) media came in the second order with tensile value of 42 ± 6 MPa, and Young’s modulus of 14 ± 3 MPa. Eventually, the BNC of the M-HS and F-HS showed comparable values for the both determinations.

**Table 4:** Tensile properties of the BNC films produced on (HS), (D-HS), (F-HS), and (M-HS) media.

Medium	Tensile strength (MPa)	Elongation at break (%)	Young’s modulus (MPa)
HS	42 ± 6	4.3 ± 2.7	14 ± 3
D-HS	57 ± 6	2.4 ± 1.1	17 ± 4
F-HS	33 ± 7	1.7 ± 0.9	10 ± 6
M-HS	38 ± 6	3.6 ± 1.8	10 ± 5

Values are presented as means ± SD.

In terms of ductility, the BNC film of the HS medium exhibited the highest ductility measured by elongation at break (4.3 ± 2.7%), followed by the film derived from M-HS (3.6 ± 1.8%); whereas, the elongation at break of films from D-HS and F-HS recorded 2.4 ± 1.1% and 1.7 ± 0.9%, respectively. The results presented a significant correspondence between the crystallinity of the BNC fibers from one side, and the tensile and Young’s modulus in the other side. This is in accordance with a previous investigation^57^, which compared the mechanical properties of the wet BNC, lyophilized BNC, and oven-dried BNC films produced by the strain *Gluconacetobacter hansenii* CGMCC 3917.

Utilizing various wastes as substrates for cultivating the BNC-producing strains will impact the physical properties of the produced BNC, where the mechanical properties for instance are substantial for exploiting BNC in food packaging and biomedical applications.

Our findings are encouraging and are consequently recommended to optimize the significant parameters for boosting BNC production by *K. saccharivorans* MD1. Moreover, some composites based on BNC could be developed, characterized, and nominated for adequate application.

## Conclusions

We isolated and identified the strain *Komagataeibacter saccharivorans* MD1 as a novel BNC producer. Furthermore, we explored the potentiality of the pretreated date fruit wastes, fig fruit wastes, and sugarcane molasses as carbon sources for BNC biosynthesis. After 168 h of static incubation, the molasses medium (M-HS) achieved the highest BNC yield and the best substrate conversion ratio. Increasing the proportions of the carbon sources reduced the BNC production, whereas utilizing low concentrations of date wastes or molasses considerably increased the BNC yield. The four BNC products showed almost the same size of nanofibers and porosity degree under FESEM analyses, while their surface roughness varied greatly; where the (HS) product record the roughest surface, while the smoothest was the (D-HS) one. XRD diffraction revealed slight variations in crystallinity indices and crystallite sizes. However, the FTIR spectra of the four sheets show identical chemical profile assigned to cellulose I. Mechanical properties and water holding capacity revealed the great versatility of the produced BNCs, which confer them the feasibility to implement in diverse applications and manifold.

## Methodology

### Screening and isolation of BNC producers

Eleven samples of garden flowers, rotten fruits, fermented foods, fermented beverage, and vinegar remnants were collected to isolate BNC producing strains. The production was carried out on Hestrin-Schramm^33^ (HS) medium consists of (g/L): D-glucose (20), peptone (5), yeast extract (5), sodium di-basic hydrogen phosphate (2.7), and citric acid (1.15). D-Glucose and citric acid were purchased from Fisher chemical; peptone, yeast extract and agar were purchased from Conda Lab; and the sodium di-basic hydrogen phosphate was provided from Sigma Aldrich and used as received. The 11 Erlenmeyer flasks were incubated at 28 °C for 10 days. The positive cultures that showed floating gel-like pellicles underwent purification procedures to isolate pure BNC-producing bacterial isolates for selecting the potent BNC producer.

### Identification of the most potent bacterial isolate

A dominant bacterial isolate that showed highest BNC yield was identified based on the morphological and physiological characters^58^. Furthermore, the bacterial cells were examined within the entangled BNC via scanning the unprocessed BNC employing FESEM. Moreover, the identification was supported with the help of a molecular tool by sequencing the 16S rRNA gene nucleotides. The PCR reaction was carried out using the universal 16S rRNA primers: 27 F Mod (5′-AGR(AG)GTTTGATCM(AC)TGGCTCAG-3′) and 1492 R Mod (5′-GGY(CT)TACCTTGTTAYGACTT-3′). The thermal cycler was set out as follows: initial denaturation at 94 °C for 5 min followed by 30 cycles of 1 min at 94 °C for further denaturation, 1 min at 55 °C for annealing, and 2 min at 72 °C for extension, and the final extension step at 72 °C for 10 min^59^. The amplified 16S rRNA gene was purified and sequenced by GenoScreen Innovative Genomics Company (Lille, France) following the protocol of Sanger approach^60^. The obtained nucleotide sequences were compared with available sequences on the National Center for Biotechnology Information GenBank (NCBI GenBank) database employing BLASTn through Basic Local Alignment Search Tool (BLAST). The comparable sequences were retrieved and multiple sequence alignment was performed by ClastalW using MEGA software (V. 6.0). Then, the phylogenetic tree was constructed adopting the Neighbor-Joining tree method, which assessed by bootstrap analysis with a value of 500^61,62^.

### Preparation of the wastes

Semi-dry date fruit (*tamer*) wastes (DFWs) and fig fruit wastes (FFWs) were collected from local markets at Alexandria governorate, while we obtained sugarcane molasses (SCM) from Al-Hawamdia Sugar Fabric., Giza, Egypt (https://www.siicegypt.com/). The seeds were excluded from date fruit wastes (DFW), while the fig fruit wastes (FFW) were first dried in a hot air oven at 70 °C for 36 h before advanced treatment.

Preparation of date and fig extracts was executed following the method described by El-Nagga & Abd El Tawab^32^ with minor amendments. Weights of 100 g of both the (DFWs) and (FFWs) were separately immersed in an equal volume of d-H_2_O for 2 h before transferring into another volume of d-H_2_O with waste: water ratio of 1:2. The soaked wastes were then homogenized using the handheld homogenizer for 10 min and transferred to a water bath at 70 °C for 1 h before double filtration by Whatman filter paper no 41. The final two syrups were collected, adjusted at an overall volume of 100 ml for each. Afterward, they were autoclaved at 121 °C for 20 min and stored in the fridge at 4 °C, labeled as extracts of date fruit wastes (E-DFW) and fig fruit wastes (E-FFW) stock solutions.

The sugarcane molasses (SCM) solution was treated following Bae & Shoda^63^ protocol of acid-heat pre-treatment. About 100 g of sugarcane molasses (SCM) was diluted by 2 folds (w/v) of d-H_2_O before centrifugation at 6000 rpm for 20 min to eliminate solid matters. The supernatant was then collected and its pH was altered to 3.0 with 4 N H_2_SO_4_, heated at 120 °C for 20 min, retained overnight at room temperature. Subsequently, the supernatant was centrifuged at 6000 rpm for 20 min and adapted to a volume of 100 ml, before storing at 4 °C as a treated sugarcane molasses (T-SCM) stock solution.

### Preparation of bacterial inocula

Inocula of isolate MD1 were prepared on (HS) medium for the whole study. Aliquots of 10 ml volume of sterile (HS) medium were prepared and inoculated by a loopful of 10 days old culture. The inoculated aliquots were incubated at 28 °C for 7 days in the dark. Upon using, all tube contents were gently mixed and the liquid phase was exploited as the inocula for all accomplished BNC biosynthesis beakers.

### Media and BNC production

In the present study, (HS) medium was the principal BNC production medium. Another three sets of (HS) media were formulated, but the supplemented glucose was substituted by (v/v) 10% of the 3 stock solutions (E-DFW, E-FFW, and T-SCM), and the prepared blends labelled as (D-HS), (F-HS), and (M-HS), respectively. The initial pH was adjusted at 6 by using 0.1 N H_2_SO_4_. The production volumes for all media were 50 ml in 250 ml beakers, which were inoculated and statically incubated in the dark at 28 °C for 7 days.

### Harvesting and purification of the produced BNC

Positive outcome of the nanocellulose pellicles could be perceived by the formation of a gel-like mat floating on the medium surface. The BNC films produced by the bacterial isolate were washed twice with boiling d-H_2_O and 0.1 N NaOH (2 times for each and 20 min for each step), and then thoroughly rinsed with d-H_2_O till reaching the neutral pH. The wet films were kept in water at 4 °C or dried at room temperature for 24 h.

### Sugar content

The total sugar content was determined by the sulfuric acid/anthrone colorimetric method described by Dubios *et al*.^64^. The total sugar contents of the prepared waste extracts were estimated, as well as the final sugar contents after the cultivation.

### Kinetics of BNC production

Efficiency of the BNC production was calculated using the following equations reported by **Gomes** and his co-workers^17^:

1 $${\rm{Substrate}}\,{\rm{conversion}}\,{\rm{ratio}}\,{\rm{a}}\,( \% )=({{\rm{S}}}_{{\rm{i}}}-{{\rm{S}}}_{{\rm{f}}})/{{\rm{S}}}_{{\rm{i}}}\times \,100$$

2 $${\rm{B}}{\rm{N}}{\rm{C}}\,{\rm{p}}{\rm{r}}{\rm{o}}{\rm{d}}{\rm{u}}{\rm{c}}{\rm{t}}{\rm{i}}{\rm{o}}{\rm{n}}\,{\rm{r}}{\rm{a}}{\rm{t}}{\rm{e}}\,{\rm{R}}\,{\mathtt{(}}{\mathtt{g}}{\mathtt{/}}{\mathtt{L}}.{\rm{H}}{\mathtt{)}}{\mathtt{=}}{\rm{M}}{\mathtt{/}}{\mathtt{(}}{\rm{V}}.{\rm{t}}{\mathtt{)}}$$

3 $${\rm{B}}{\rm{N}}{\rm{C}}\,{\rm{p}}{\rm{r}}{\rm{o}}{\rm{d}}{\rm{u}}{\rm{c}}{\rm{t}}{\rm{i}}{\rm{o}}{\rm{n}}\,{\rm{y}}{\rm{i}}{\rm{e}}{\rm{l}}{\rm{d}}\,{\rm{Y}}\,({\rm{ \% }})=({\rm{M}}/{\rm{V}})/({{\rm{S}}}_{{\rm{i}}}-{{\rm{S}}}_{{\rm{f}}})\times \,100$$

where (S_i_) is the initial substrate concentration (g/L), (S_f_) is the final substrate concentration (g/L), (M) is the amount of the produced BNC (g), (V) is the production volume (L), and (t) is the cultivation time period (h).

### Time course of the BNC production on the utilized four media

The production of BNCs on the four formulations was conducted for an incubation period of 168 h to explore the production behavior, time point of maximal BNC production, and the pH fluctuation for the utilized BNC-producing strain on each medium. The obtained films were washed and dried as mentioned above. The pH values were measured for the whole harvested liquid cultures; subsequently, means and standard deviations (SD) were plotted versus time (h).

### Influence of carbon source concentration on BNC productivity

The impact of the different carbon source concentrations on the BNC productivity was examined. The same media formulations; (D-HS), (F-HS), and (M-HS) were prepared in the ordinary proportion (v/v) 10%, in addition to the proportions of 5, 20, and 30% for each waste. The inocula proportion fixed at (v/v) 10% for all replicates, and they were statically incubated in the dark at 28 °C for 7 days.

### Physical characterization of the BNC produced on HS and the three wastes media field-emission scanning electron microscope (FESEM) analysis

We investigated the produced BNC films using field emission scanning electron microscope (FESEM) by mounting a piece of about 3 × 3 mm on a SEM metal holder and sputter the sample surface by gold nanoparticles for 2 min. The sputtered samples were then scanned by Quanta^TM^ Field emission gun (FEG 250) high resolution scanning electron microscope (SEM) at a voltage of 5 kV using the Everhart-Thornley Detector (ETD) at high vacuum.

### Water holding capacity (WHC) and water release rate (WRR)

To evaluate water holding capacity (WHC) of the four BNC products, never dried samples were extracted from storing and the excess water was blotted by a paper towel. The samples were weighed (*W*_wet_), and then left to dry in room temperature for 48 h. Afterwards, the samples were dried at 50 °C for 12 h to evaporate any humidity remnants, before determining the final weight of samples (*W*_dry_). The water holding capacity for the BNC sample can be estimated using Eq. 4 as follow:

4 $${\rm{WHC}}\,({\rm{g}}/{\rm{g}})={W}_{{\rm{wet}}}({\rm{g}})/{W}_{{\rm{dry}}}({\rm{g}})$$

With regard to water release rate (WRR), never dried BNC samples were weighed (*W*_wet_), and then left in room temperature with frequent weighing of the samples every 4 h. The weights were plotted as percentages of the *W*_wet_ versus time (h).

### Analysis by atomic force microscopy (AFM)

The BNCs generated on the four media were analyzed using atomic force microscopy AFM (Keysight 5500LS) to characterize surface topography. The tapping mode was employed for imaging an area of 30 ×30 µm for each sample. The root mean square roughness RMS (Rq) was applied to compare surface roughness of the four BNC products. (Rq) is defined as the mean squared absolute values of surface roughness profile (µm) representing more sensitive parameter to surface peaks and valleys due to the squaring of amplitudes in its calculation^65^.

### X-ray diffraction (XRD) analysis

Structural properties of the obtained BNCs from the four media were examined employing X-ray Diffractometer (labX XRD-6100, Shimadzu, Japan). The patterns were recorded at the CuKα radiation wavelength (λ = 1.54 Å), generated at a voltage of 40 kV and a filament emission of 30 mA. BNC samples were scanned at 2Ɵ range of 5–80 degrees at a scan speed of 0.5° min^−1^. Crystallinity was calculated through the following equation:

5 $${\rm{CrI}}\,( \% )={[I}_{(200)}-{{\rm{I}}}_{(\text{am})}/{{\rm{I}}}_{(200)}]\ast 100$$

where I_(200)_ is the intensity of the peak at 2Ɵ = 22°, I_(am)_ is the background height between the peaks 2Ɵ = 22° and 2Ɵ = 16°.

The crystallite size was estimated using Scherrer’s equation:

6 $${\rm{CrS}}=k{\rm{\lambda }}/({\rm{\beta }}\,\cos \,\Theta )$$

where (k) is the dimensionless Scherrer constant = 0.9, (λ) is the X-ray wavelength, (β) is the peak full width at half maximum in radians, and (Ɵ) is the diffraction angle in radians.

Bragg’s equation was used to determine the atomic inter-planar spacing (d) as following:

7 $${\rm{d}}=n{\rm{\lambda }}/2\,\sin \,\Theta $$

where (n) is the order of the peak plane^43^.

### Analysis by Fourier transform infrared (FTIR) spectrophotometry

The FTIR (FTIR-8400 S, Shimadzu, Japan) was applied to compare the functional groups of the processed BNC films from the utilized four media at spectra range 4000-400 cm^−1^ at 4 cm^−1^ resolution for 32 scans prior to the Fourier transformation.

### Mechanical properties determination

The mechanical properties of the four BNC films were characterized by a universal testing machine (AG-1S, SHIMADZU, Japan). Each film was cut into rectangle with dimension of 20 ×50 mm, and the thickness was then gauged by (Sealey AK9635D) digital micrometer. The cell preload was 5 N and testing speed of 10 mm/min was applied to reach a constant strain rate. Young’s modulus, tensile strength and elongation at break (%) were calculated from the stress-strain data through exponent software.

### Statistical analysis

All investigations were performed in six replicates, and the results were statistically analyzed using GraphPad Prism software (Version 7). The data were analyzed employing one-way and two-way analysis of variance (ANOVA) with the Tukey’s test for multiple comparisons^66^. The multiple comparisons were carried out based on the values, which were expressed by means ± SD of each group. The significant values were determined at *P-value* < 0.05, whereas the high significant values were considered at *P-value* < 0.01, and *P-value* < 0.001 using n = 6.

## Supplementary information

Supplementary information.
