# Supplementary material for: Bacterial nanocellulose from agro-industrial wastes: low-cost and enhanced production by Komagataeibacter saccharivorans MD1
Source: Sci Rep. 2020 Feb 26;10:3491. doi: 10.1038/s41598-020-60315-9 (PMC7044201; doi:10.1038/s41598-020-60315-9)
Supplement: Supplementary file 1 — Supplementary information. [file 41598_2020_60315_MOESM1_ESM.docx]

**Supplementary information**

**Bacterial nanocellulose from agro-industrial wastes: low-cost and enhanced production by *Komagataeibacter saccharivorans* MD1**

**Deyaa Abol-Fotouh^1,*^, Mohamed A. Hassan^2,*^, Hassan Shokry^1,3^, Anna Roig^4^, Mohamed S. Azab^5^ and Abd El-Hady B. Kashyout^1,*^**

^1^Electronic materials Researches Department, Advanced Technology and New Materials Research Institute, City of Scientific research and technological applications (SRTA-City), New Borg El-Arab City, P.O. Box: 21934 Alexandria, Egypt

^2^Protein Research Department, Genetic Engineering and Biotechnology Research Institute (GEBRI), City of Scientific research and technological applications (SRTA-City), New Borg El-Arab City, P.O. Box: 21934 Alexandria, Egypt

^3^Environmental Engineering Department, Egypt-Japan University of Science and Technology, New Borg El-Arab City, Alexandria, Egypt.

^4^Institute of Materials Science of Barcelona (ICMAB-CSIC), Campus of the UAB, Bellaterra, Spain.

^5^Department of Botany & Microbiology, Faculty of Science, Al-Azhar University, Cairo, Egypt.

**^*^Corresponding Author:**

1. **Mohamed A. Hassan**

E-mail: madel@srtacity.sci.eg

Tel: +2034593422

1. **Deyaa Abol-Fotouh**

E-mail: dabolfotouh@gmail.com

Tel: +2034593414

1. **Abd El-Hady B. Kashyout**

E-mail: akashyout@srtacity.sci.eg

Tel: +2034593414

| **Characteristics** | **Isolate (MD1)** |
| --- | --- |
| Gram stain | Gram -ve |
| Cell shape | Rod shaped |
| Motility | No |
| Colony color | Beige, opaque |
| Colony shape | Circular |
| Colony margin | Entire |
| Colony elevation | Convex |
| Growth anaerobic | -ve |
| Growth without acetic acid | +ve |
| Growth on |  |
| Glutamate agar | +ve |
| Mannitol agar | +ve |
| Production of water-soluble brown pigment | -ve |
| Production of acids from: |  |
| D-glucose | +ve |
| D-arabinose | +ve |
| D-ribose | +ve |
| D-galactose | +ve |
| D-fructose | +ve |
| Lactose | -ve |
| Maltose | -ve |
| Sucrose | -ve |

**Table S1.** Morphological and physiological characteristics of bacterial isolate MD1.


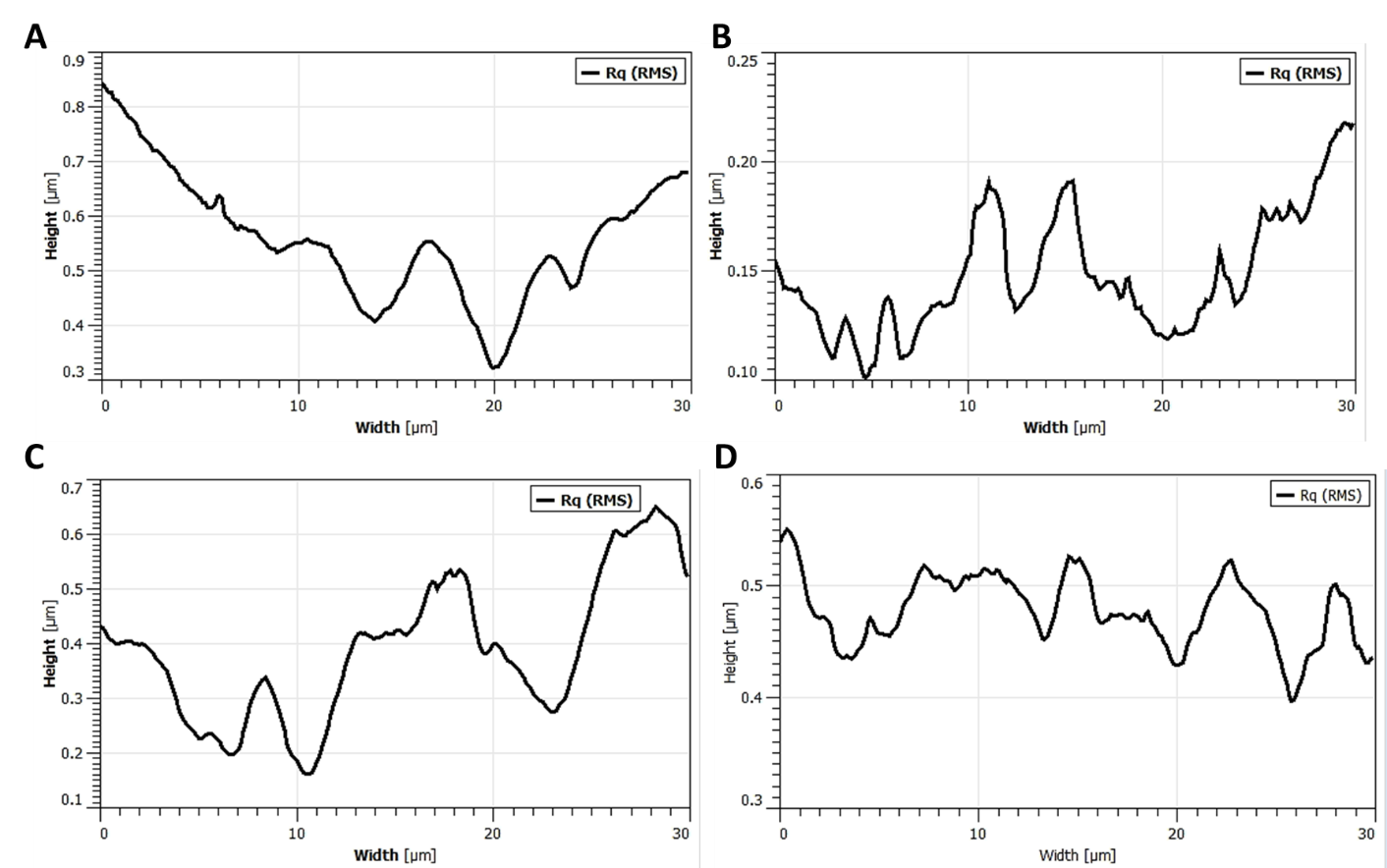


**Figure S1.** AFM 3D imaging and the corresponding Fourier transform patterns of the surfaces BNC produced on (A) HS, (B) D-HS, (C) F-HS, and (D) M-HS Media.

|  | **HS** | **D-HS** | **F-HS** | **M-HS** |
| --- | --- | --- | --- | --- |
| **Rq (µm)** | 0.67 | 0.19 | 0.44 | 0.48 |
| **Rsk** | -0.45 | 0.20 | 0.43 | 0.44 |
| **Rku** | 2.50 | 2.48 | 3.09 | 2.37 |
| **Rp (µm)** | 1.47 | 0.77 | 1.64 | 1.62 |
| **Rv (µm)** | 1.88 | 0.59 | 1.15 | 0.94 |
| **Rz (µm)** | 3.35 | 1.37 | 2.78 | 2.56 |
| **Ra (µm)** | 0.55 | 0.16 | 0.35 | 0.40 |

**Rq**: root mean square roughness; **Rsk**: skewness; **Rku**: kurtosis; **Rp**: maximum height of peaks; **Rv**: maximum depth of valleys; **Rz**: ten-points height; **Ra**: arithmetic average height.

**Table S2.** Extra data expressing the surface roughness of the BNC fabricated on HS, D-HS, F-HS, and M-HS media.
